# Propolis nanoparticle enhances the potency of antimicrobial photodynamic therapy against *Streptococcus mutans* in a synergistic manner

**DOI:** 10.1038/s41598-020-72119-y

**Published:** 2020-09-23

**Authors:** Shima Afrasiabi, Maryam Pourhajibagher, Nasim Chiniforush, Abbas Bahador

**Affiliations:** 1 Department of Microbiology, School of Medicine, Tehran University of Medical Sciences, Tehran, Iran; 2 Dental Research Center, Dentistry Research Institute, Tehran University of Medical Sciences, Tehran, Iran; 3 Dental Implant Research Center, Dentistry Research Institute, Tehran University of Medical Sciences, Tehran, Iran; 4 Oral Microbiology Laboratory, Department of Microbiology, School of Medicine, Tehran University of Medical Sciences, Keshavarz Blvd, 100 Poursina Ave., 14167-53955 Tehran, Iran

**Keywords:** Microbiology, Medical research

## Abstract

Less invasive removal approaches have been recommended for deep caries lesions. Antimicrobial photodynamic therapy (aPDT) and propolis nanoparticle (PNP) are highlighted for the caries management plan. Evidence is lacking for an additive effect of combination PNP with photosensitizer (PS) in aPDT. This study aimed to investigate the individual and synergistic effects of chlorophyllin-phycocyanin mixture (PhotoActive^+^) and toluidine blue O (TBO) as PSs in combination with PNP in the aPDT process (aPDT^plus^) against major important virulence factors of *Streptococcus mutans.* Following characterization, biocompatibility of the PSs alone, or in combination with PNP were investigated on human gingival fibroblast cell. The in vitro synergy of PhotoActive^+^ or TBO and PNP was evaluated by the checkerboard method. The bacteria's virulence properties were surveyed in the presence of the PSs, individually as well as in combination. When the PSs were examined in combination (synergistic effect, FIC Index < 0.5), a stronger growth inhibitory activity was exhibited than the individual PSs. The biofilm formation, as well as genes involved in biofilm formation, showed greater suppression when the PSs were employed in combination. Overall, the results of this study suggest that the combination of PhotoActive^+^ or TBO with PNP with the least cytotoxicity effects and the highest antimicrobial activites would improve aPDT outcomes, leading to synergistic effects and impairing the virulence of *S. mutans*.

## Introduction

The complete removal of carious dentin close to the dental pulp presents a serious challenge^1^. Avoiding pulp exposure, elimination of residual microorganisms into the cavities, maintenance of functional teeth and mineral structures for longer periods are the major objectives of successful caries treatment. Selective removal of dental caries (SRDC) has been supported for this purpose^2,3^. *Streptococcus mutans* is well documented as a main etiological agent in caries initiation^4^. Among the virulence factors of *S. mutans*, glucosyltransferases (Gtfs) and fructosyltransferase (Ftf) enzymes are especially important because of their performance, which lead to synthesis of exopolysaccharides (EPS)^5^. *S. mutans* possess three distinct Gtfs, encoded by *gtfB*, *-C*, and *-D* and a single *ftf gene,* expressing Ftf*.* Among related genes, *gtfB* and *gtfC* produce water-insoluble glucans that is essential for tenacious adherence of S*. mutans* to the surface of tooth^5,6^. Ftf can catalyze the synthesis of fructan and glucose from sucrose. In addition, it plays a role as an extracellular carbohydrate reservoir^7^.

Antimicrobial photodynamic therapy (aPDT) as a complementary step of SRDC can be a promising candidate instead of conventional methods such as mechanical debridement or application of adjunctive antiseptics^2,8^. Generally, aPDT is a noninvasive technique that has shown the potential to help treat localized microbial infections^8^. Several studies have reported different degrees of bacterial biofilms reduction of S*. mutans* by utilizing different photosensitizers (PSs)^2,9–13^.

Photosensitization using plant-based PSs or photoactive food additive demonstrated beneficial efficacy against microbial infections^14^. PhotoActive + is a mixture of chlorophyllin (CHL) and phycocyanin (PC). CHL is a photosynthetic green pigment of chlorophyll. It presents in dietary supplements^15^. Also, PC has been used mainly as a food pigment. It is a new class of fluorescent dye which found in *Spirulina platensis*^16^. Many pharmacological activities of CHL and PC, such as anti-inflammatory, anticancer, and antimicrobial functions have been revealed^15,17^. Toluidine blue O (TBO) is known to be a cationic dye that documented as potential PS, target the cell membrane and is bactericidal for multiple species such as *Streptococci*^10^.

Overall, aPDT should be taken into consideration because of their advantages, including the elimination of resistant microorganisms, few complications, improved selectivity and rapid time of action^18^. Even though locally applied aPDT avoids systemic adverse effects, high concentrations of PSs and high-energy doses of light are usually required to completely eliminate infectious bacteria^19^. Moreover, the use of dye might cause tooth and restoration staining, thus critically affecting its clinical application^20^. To overcome such problems, aPDT can be combined with other therapeutic modalities and improved overall results while reducing individual concentrations of PSs^19^.

Propolis as a natural product collected by bees from various parts of plant sources is known to possess pharmacological activities, including anti-inflammatory, anticancer, antioxidant, antifungal, antiviral and antimicrobial effects^21^. Propolis nanoparticles (PNPs) are more effective in treatment because particle size decreases and the surface/volume ratio become too large. This property makes them highly reactive and can overcome some of the drawbacks in raw propolis^22^. Generally, NPs are applied to increase the delivery of PS to their target in order to improve the effectiveness of aPDT ^11^.

No research has been conducted on combining the PNP with PS to obtain a synergistic effect and improved aPDT outcomes without the need to administrate maximal concentrations of the PSs. Therefore, the purpose of this study was to explore a combinational approach during aPDT via the combination of PhotoActive^+^ or TBO and PNP to further suppress cariogenic virulence factors of *S. mutans*. It was hypothesized that this approach will potentiate the efficacy of aPDT.

## Results

### MICs of PSs

PhotoActive^+^ (625–1,250 μg/mL) and TBO (12.5–100 μg/mL) significantly reduced *S. mutans* growth when compared to untreated bacteria (control; *P* < 0.05). Lower concentrations of PhotoActive^+^ (2.4–312.5 μg/mL) and TBO (0.19–6.25 μg/mL) also affected *S. mutans* growth, but this was not statistically significant (*P* > 0.05). Therefore, the MIC of PhotoActive^+^ and TBO were 625 and 12.5 μg/mL, respectively.

### Doses of aPDT contributing to sub-significant reduction of CFU/ mL

Based on the results, there was a marked reduction against *S. mutans* cells growth by the PhotoActive^+^ (312.5 μg/mL at a fluency of 103.12 J/ cm^2^) and TBO-aPDT (12.5 μg/mL at a fluency of 68.75 J/cm^2^) compared with the control (*P* < 0.05). Whereas there was no significant bacterial CFU/mL reduction in the presence of 156.2 μg/mL PhotoActive^+^ plus diode laser at fluency of 103.12 J/ cm^2^ and 6.25 μg/mL TBO plus diode laser at fluency of 68.75 J/cm^2^ in comparison to the control group (*P* > 0.05). Taken together, the sub-significant reduction dose of aPDT against CFU/mL of *S. mutans* cells was determined with 156.2 μg/mL of PhotoActive^+^ at a fluency of 103.12 J/cm^2^, and 6.25 μg/mL of TBO at a fluency of 68.75 J/cm^2^.

### Characterization of PNP

**Figure 1:**
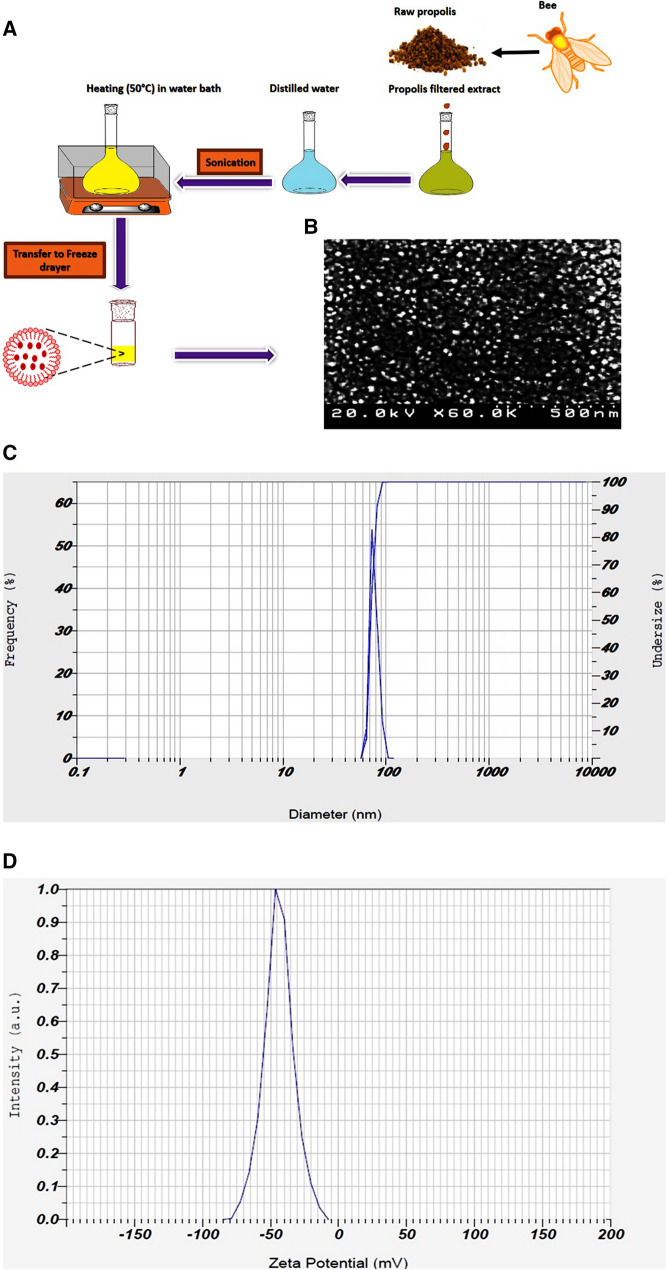
Characterization of PNP. (**A**) Schematic representation of PNP preparation; (**B**) FE-SEM image of the PNP; (**C**) size distributions of the PNP; (**D**) the zeta potential of PNP. *PNP* propolis nanoparticle.

Figure 1A shows the PNP preparation. The Fe-SEM image reveals that the PNP morphology is nearly spherical (Fig. 1B). Figure 1C indicates the DLS data of the PNP at 25 °C. As shown, the PNP was a nano-sized particle around 70- to 75- nm in diameter. Zeta potential of PNP showed a negative surface charge value (− 44 mV) which was sufficiently high to avoid NPs aggregation (Fig. 1D). This value represents a stable and dispersed suspension of NPs that there is no tendency to form aggregates in a short period of time.

### MIC of PNP

PNP (25–50 mg/mL) inhibited *S. mutans* growth when compared to control (*P* < 0.05). But lower concentrations of PNP (12.5–0.09 mg/mL) did not inhibit *S. mutans* growth (*P* > 0.05). So, the MIC of PNP was 25 mg/ml, which inhibited the growth of *S. mutans* cells.

### Time-kill assay

**Figure 2:**
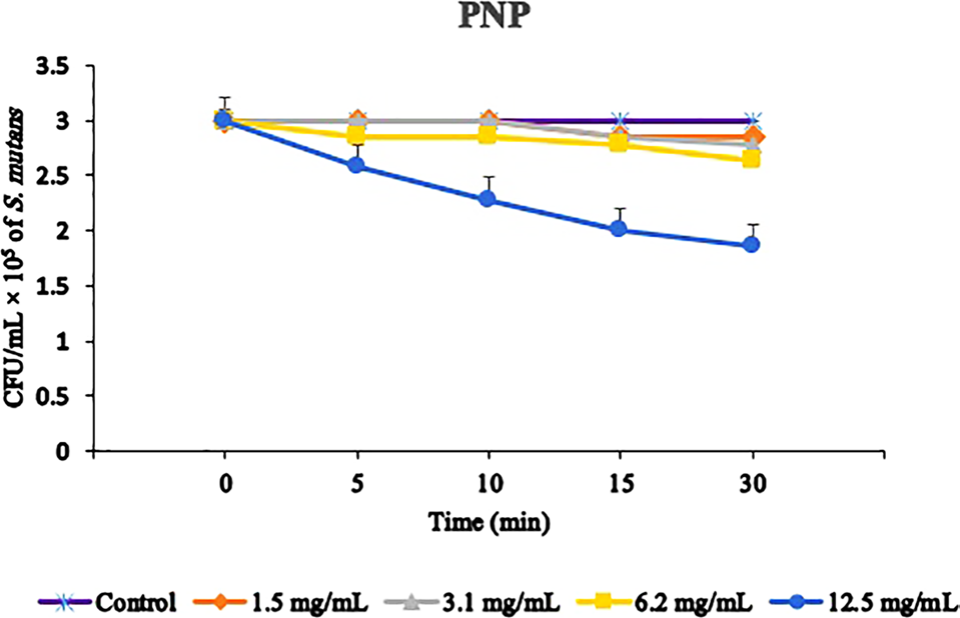
Time–kill assay of PNP. The suspensions of *Streptococcus mutans* were submitted to incubation with PNP for different time periods. Error bars represent standard deviation. *PNP* propolis nanoparticle.

According to Fig. 2, PNP in sub-MIC concentrations (1.5, 3.1, 6.2 and 12.5 mg/mL) with up to 30 min of incubation did not display a significant CFU/mL reduction among the incubation times (*P* > 0.05). Therefore, 5 min of incubation time was used, which is the usual pre-incubation time for most PSs.

### The synergistic effect of PhotoActive^+^ or TBO in combination with PNP

**Figure 3:**
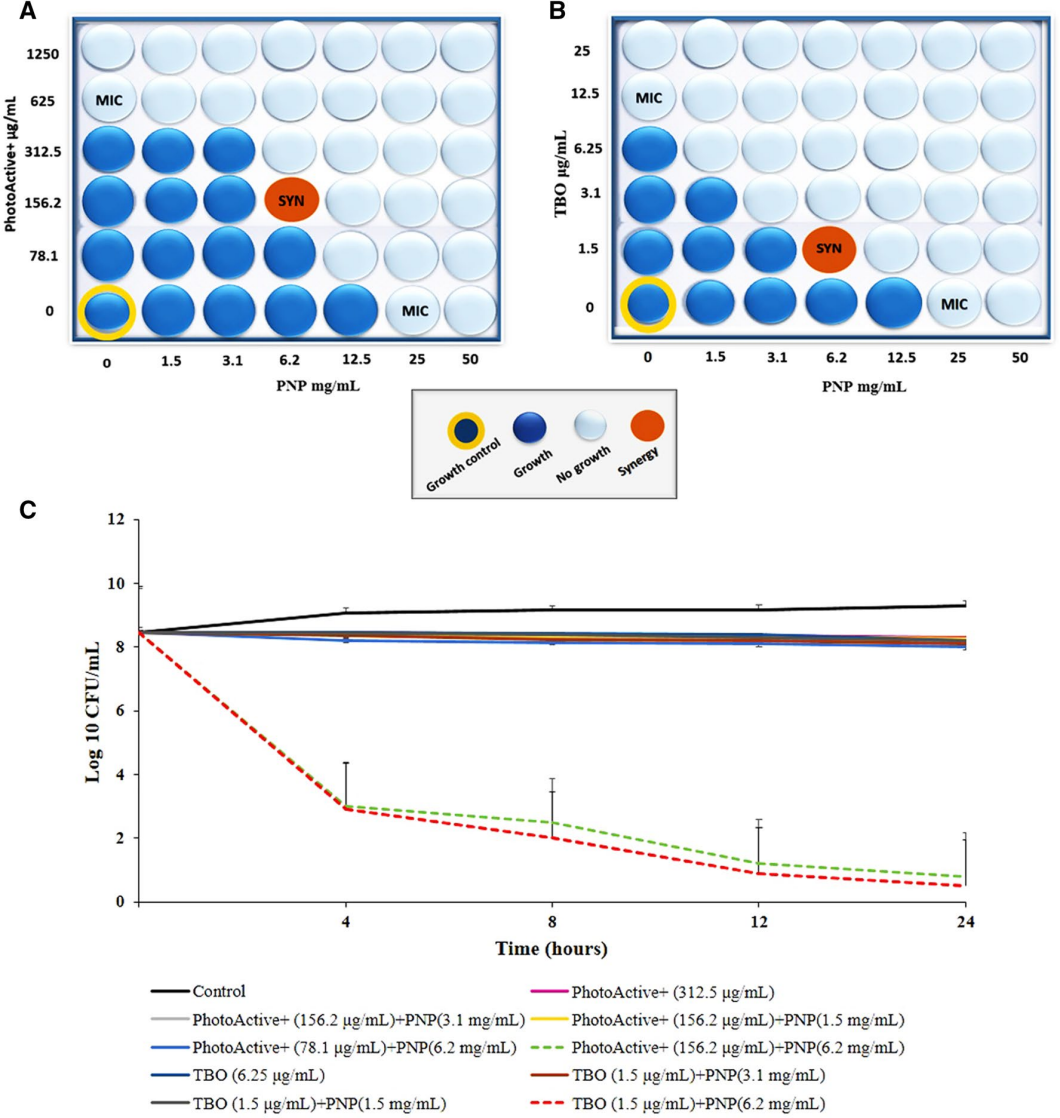
Synergistic effect of PhotoActive^+^ or TBO with PNP. Schematic checkerboard of *Streptococcus mutans* growth inhibition with varying concentrations of PNP and (**A**) PhotoActive^+^ or (**B**) TBO. Each well represents a value; (**C**) time-kill and synergism studies of PNP and PhotoActive^+^ or TBO against *Streptococcus mutans*. Error bars represent standard deviation. *PNP* propolis nanoparticle, *TBO* toluidine blue.

Combining PhotoActive^+^ or TBO with PNP showed a synergistic antibacterial effect. Both MIC of PhotoActive^+^, TBO and MIC of PNP were used to calculate FICI. According to the checkerboard-method results (Fig. 3A,B), PhotoActive^+^ and TBO in a concentration of 156.2 and 1.5 μg/mL, respectively, with PNP at a concentration of 6.2 mg/mL showed a synergistic effect as the FICI value dropped below 0.5.

### The time kill effect of PhotoActive^+^ or TBO in combination with PNP

A combination therapy of PNP (at 6.2 mg/mL) and PhotoActive^+^ or TBO (at 156.2 and 1.5 μg/mL, respectively) produced more than 3 log_10_ higher killing efect at 24 h post-treatment compared with each agent alone. The combination of low dose PNP (at 1.5 and 3.1 mg/L) and PhotoActive^+^ (at 156.2 and 78.1 μg/mL) or TBO (at 1.5 μg/mL) showed no synergistic effects compared with each agent alone (Fig. 3C).

### Combination treatment (aPDT^plus^)

**Figure 4:**
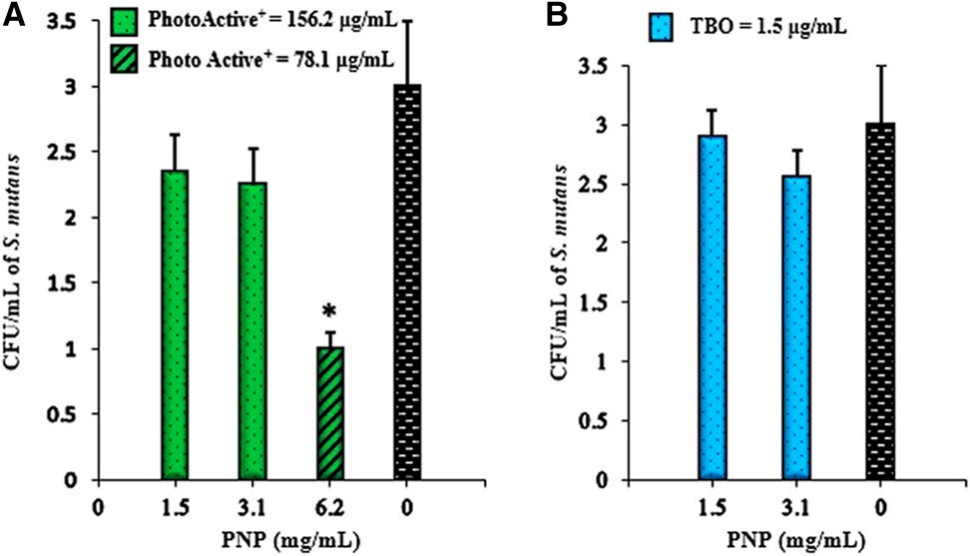
Combination treatment against *Streptococcus mutans*. The suspension of *Streptococcus mutans* was submitted to aPDT combined to the PNP at sub-MIC level (aPDT^plus^). (**A**) PhotoActive^+^ -aPDT^plus^: 156.2 and 78 μg/mL of PhotoActive^+^  + 103.12 J/cm^2^; (**B**) TBO-aPDT^plus^: 1.5 μg/mL of TBO + 68.75 J/cm^2^. Error bars represent standard deviation. * *P* < 0.05. *aPDT* antimicrobial photodynamic therapy, *TBO* toluidine blue.

PhotoActive^+^ mediated aPDT using 78.1 μg/mL of PhotoActive^+^ in combination with PNP in a concentration of 6.2 mg/mL at a fluency of 103.12 J/cm^2^ showed a significant reduction against *S. mutans* growth when compared to the control group (*P* < 0.05; Fig. 4A). Treatment at 156.2 μg/mL of PhotoActive^+^ and 1.5 μg/mL of TBO in combination with PNP of a concentration of 1.5 and 3.1 mg/mL with irradiation at a fluency of 103.12 J/cm^2^ and 68.75 J/cm^2^, respectively did not exhibit a significant CFU/mL reduction (*P* > 0.05; Fig. 4A,B). Therefore, 156.2 μg/mL of PhotoActive^+^ and 1.5 μg/mL of TBO in combination with PNP in concentration of 3.1 mg/mL at a fluency of 103.12 J/cm^2^ and 68.75 J/cm^2^, respectively, were determined as the aPDT^plus^ in a combination process.

### Cytotoxicity assay

**Figure 5:**
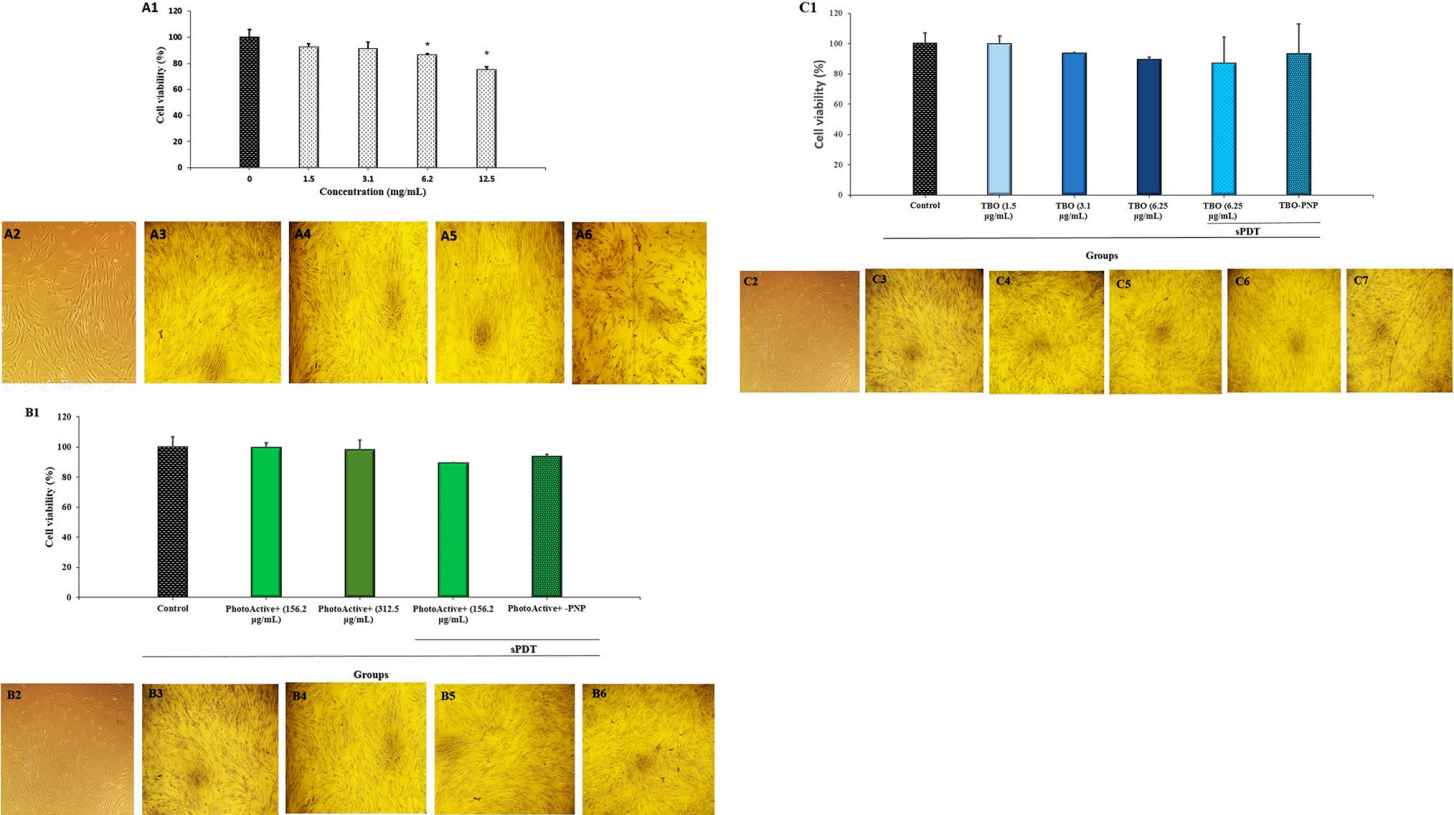
(**A1**) Cell viability assay of HGF cells exposed to PNP at different concentrations. (**A2**) Control group (**A3**); representative photograph of morphological changes for HGF cells in presence of PNP at 1.5 mg/mL (**A4**); representative photograph of morphological changes for HGF cells in presence of PNP at 3.1 mg/mL (**A5**); representative photograph of morphological changes for HGF cells in presence of PNP at 6.2 mg/mL (**A6**), representative photograph of morphological changes for HGF cells in presence of PNP at 12.5 mg/mL. (**B1**) Cell viability assay of HGF cells exposed to PhotoActive^+^, PhotoActive^+^-aPDT and aPDT^plus^; (**B2**) control group; (**B3**) representative photograph of morphological changes for HGF cells in presence of PhotoActive^+^ at 156.2 μg/mL; (**B4**) representative photograph of morphological changes for HGF cells in presence of PhotoActive^+^ at 312.5 μg/mL; (**B5**) Representative photograph of morphological changes for HGF cells in presence of PhotoActive^+^ -aPDT: 156.2 μg/mL of PhotoActive^+^  + diode laser with energy density of 103.12 J/cm^2^; (**B6**) Representative photograph of morphological changes for HGF cells in presence of PhotoActive^+^-aPDT^plus^: 156.2 μg/mL of PhotoActive^+^  + 3.1 mg/mL of PNP + diode laser with energy density of 103.12 J/cm^2^. (**C1**) Cell viability assay of HGF cells exposed to TBO, TBO-aPDT and aPDT^plus^; (**C2**) control group; (**C3**) Representative photograph of morphological changes for HGF cells in presence of TBO at 1.5 μg/mL; (**C4**) Representative photograph of morphological changes for HGF cells in presence of TBO at 3.1 μg/mL; (**C5**) Representative photograph of morphological changes for HGF cells in presence of TBO at 6.25 μg/mL; (**C6**) Representative photograph of morphological changes for HGF cells in presence of TBO-aPDT: 6.25 μg/mL of TBO + diode laser with energy density of 68.75 J/cm^2^; (**C7**) Representative photograph of morphological changes for HGF cells in presence of TBO-aPDT^plus^: 1.5 μg/mL of TBO + 3.1 mg/mL of PNP + diode laser with energy density of 68.75 J/cm^2^. *PNP* propolis nanoparticle, *HGF* human gingival fibroblast, *TBO* toluidine blue.

The HGF cells were treated with different concentrations of PNP, PSs alone (concentration is shown in Fig. 5A1,B1,C1), and also either aPDT and aPDT^plus^. As it can be seen in Fig. 5A1, the cell viability did not affect significantly in low concentration of PNP up to 3.1 mg/mL (*P* > 0.05) and by increasing the concentration (6.2 and 12.5 mg/mL) the cell viability decreased (*P* < 0.05). In addition, the cell viability does not have significant changes after treatment with PhotoActive^+^ or TBO alone and also either PhotoActive^+^ or TBO-aPDT and PhotoActive^+^ or TBO aPDT^plus^ in the studied concentration range when compared with the control (Fig. 5B1,C; *P* > 0.05). The results of MTT assay with more than 75% cell viability, suggesting that the compounds tested in the working concentration range had a good biocompatibility with inert behavior. It is clear that the cells treated with PSs alone and also either aPDT and aPDT^plus^ and PNP at different concentrations do not display any appreciable morphological changes when compared with the control cells.

### Hemolysis assay

**Figure 6:**
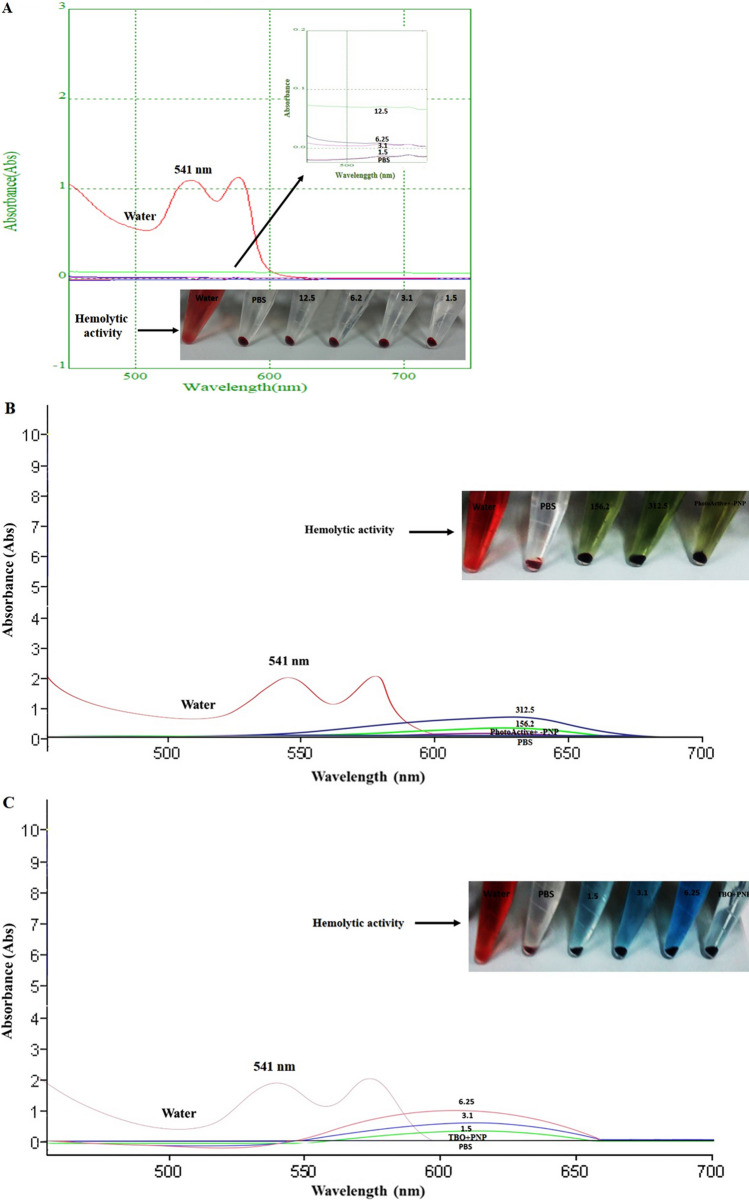
(**A**) The hemolysis assay of HRBSs exposed to PNP at different concentrations (1.5, 3.1, 6.2, and 12.5 mg/mL). Water and PBS were used as positive and negative control, respectively. The bottom-right of the curve insets demonstrate the image of HRBCs exposed to water, PBS, and PBS containing PNP at different concentrations. The upper-right of the curve insets exhibit the enlarged UV–Vis spectra shown. (**B**) The hemolysis assay of HRBSs exposed to PhotoActive^+^ at different concentrations (156.2 and 312.5 μg/mL) and PhotoActive^+^ (156.2 μg/mL) in combination with PNP (3.1 mg/mL). Water and PBS were used as positive and negative control, respectively. The upper-right of the curve insets demonstrate the image of HRBCs exposed to water, PBS, and PhotoActive^+^ at different concentrations as well as PhotoActive^+^ in combination with PNP. (**C**) The hemolysis assay of HRBSs exposed to TBO at different concentrations (1.5, 3.1 and 6.25 μg/mL) and TBO (1.5 μg/mL) in combination with PNP (3.1 mg/mL). Water and PBS were used as positive and negative control, respectively. The upper-right of the curve insets demonstrate the image of HRBCs exposed to water, PBS, and TBO at different concentrations as well as TBO in combination with PNP. *PNP* propolis nanoparticle, *HRBSs* human red blood cells, *TBO* toluidine blue.

The hemolytic activity of PNP is shown in Fig. 6A. It can be seen from the photograph that PNP exhibited good hemocompatibility, similar to the PBS as the negative control. The hemolysis percentages of the PNP in the studied concentration range (1.5, 3.1, 6.2, and 12.5 mg/mL) were 0.1, 0.2, 0.4, and 0.8%, respectively. Moreover, the blood compatibility was confirmed for each PS alone as well as in combination with PNP in the studied concentration range. As can be observed in Fig. 6B,C, both target PSs alone, and in combination with PNP exhibited excellent hemocompatibility (hemolysis percentages: all close to zero percent), similar to that of PBS. In contrast, the HRBCs exposed to water display apparent hemolysis behavior. The findings suggest good hemocompatibility for the compounds tested according to the standard acceptance value (< 5%).

### aPDT and aPDT^plus^ reduced *S. mutans* biofilm formation ability

**Figure 7:**
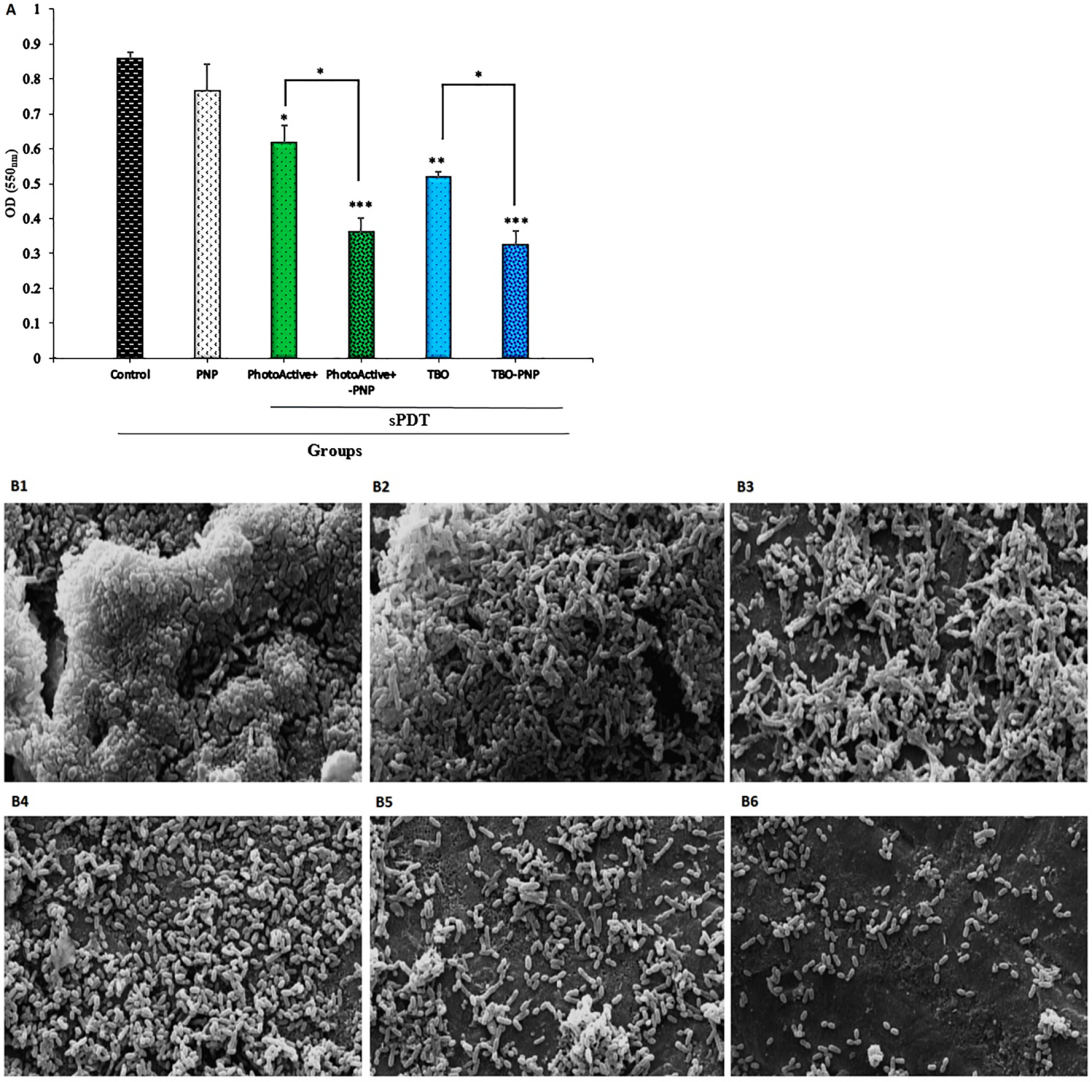

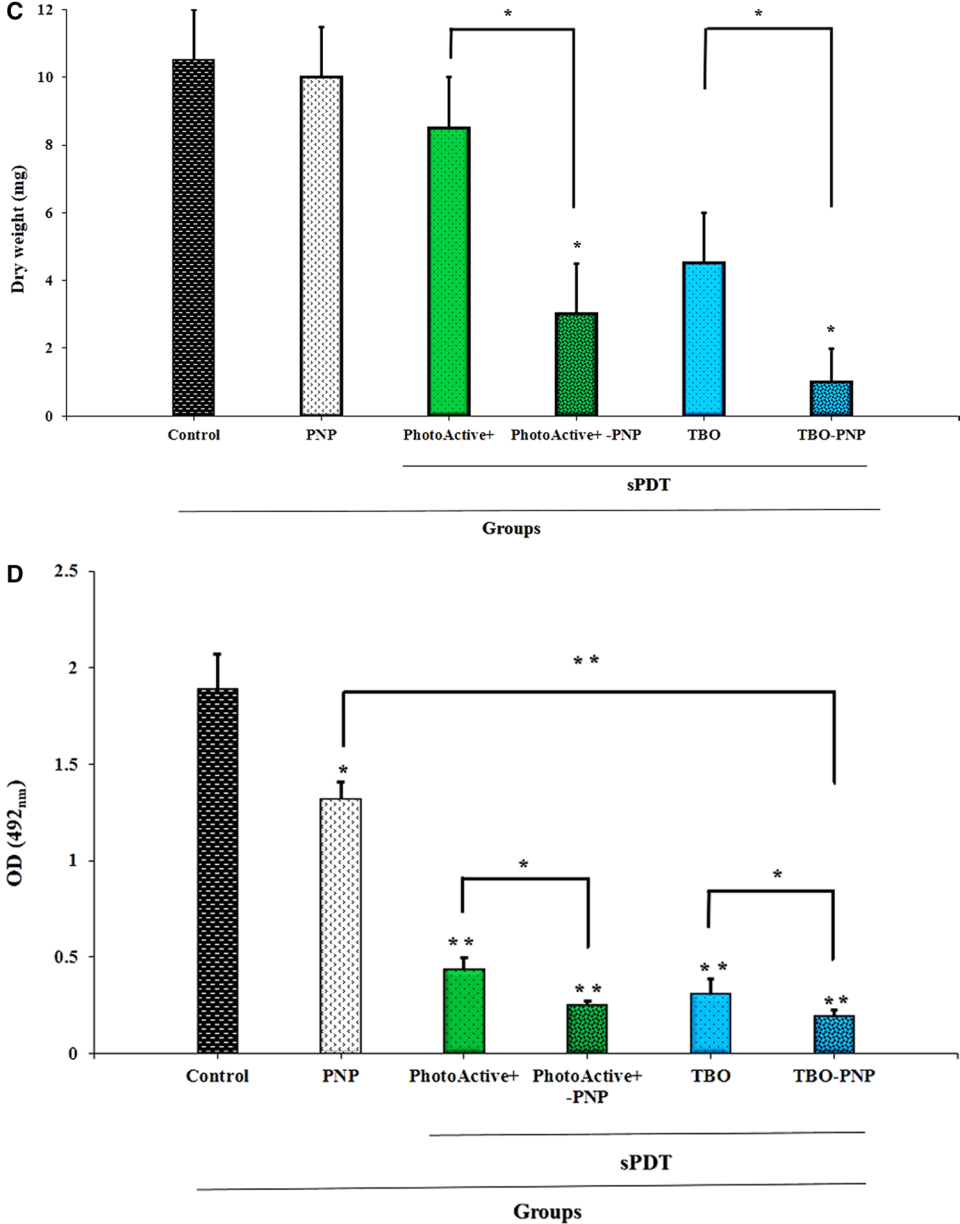
(**A**) The effect of the PNP, aPDT and aPDT^plus^ on biofilm formation of *Streptococcus mutans* determined by crystal violet assay at 550 nm. Error bars represent standard deviation. *p* < 0.05, < 0.01 and < 0.001 are shown by *, ** and ***, respectively. (**B1**–**B6**)**.** Imaging of *Streptococcus mutans* biofilms using Fe-SEM; (**B1**) Untreated biofilm control; (**B2**) biofilm treated with PNP; (**B3**) biofilm treated with PhotoActive^+^-aPDT; (**B4**) biofilm treated with TBO-aPDT; (**B5**) biofilm treated with PhotoActive^+^-aPDT^plus^; (**B6**) biofilm treated with TBO-aPDT^plus^. Fe-SEM: field emission scanning electron microscopy; PNP: propolis nanoparticle; TBO: toluidine blue. Magnification, 3,000 × . (**C**) The effect of the PNP, aPDT and aPDT^plus^ on the biomass (dry weight) in the *S. mutans* biofilm compared with control. Error bars represent standard deviation. *p* < 0.05 and < 0.01 are shown by * and **, respectively. PNP: propolis nanoparticle. (**D**) The effect of the PNP, aPDT and aPDT^plus^ on the metabolic activity of *Streptococcus mutans* determined by XTT reduction assay at 492 nm. Error bars represent standard deviation. *p* < 0.05 and < 0.01 are shown by * and **, respectively. *PNP* propolis nanoparticle.

The biofilm formation ability of *S. mutans* decreased impressively after treatment with PhotoActive^+^- and TBO-aPDT^plus^ at desired concentrations. The results of this study demonstrated that PhotoActive^+^- and TBO-aPDT prevented 28.00 and 39.68% of biofilm formation of *S. mutans* (*P* = 0.012 and 0.002, respectively), suggesting that PhotoActive^+^- and TBO-aPDT is an inhibitor of *S. mutans* biofilm. Biofilm formation of *S. mutans* was not statistically reduced with PNP alone at sub-MIC level (10.74%; *P* = 0.36). On the other hand, when the bacterial cells treated with PhotoActive^+^- and TBO-aPDT^plus^, a significant decrease in biofilm formation was observed (58.00 and 62.32%, respectively; both *P* = 0.00). Overall, the use of PhotoActive^+^- or TBO-aPDT^plus^ resulted in OD values that were significantly lower than the values obtained from the PhotoActive^+^- or TBO-aPDT (Fig. 7A).

### Observation of treated biofilm by Fe-SEM

The effect of PNP, aPDT and aPDT^plus^ on the *S. mutans* biofilm was assessed*.* The cell structure of the treated and untreated cells was investigated using Fe-SEM and results are shown in Fig. 7B1–B6. The untreated bacterial cells of *S. mutans* exhibited their original shape with clusters of cells (Fig. 7B1). As shown in Fig. 7B2, the reduction induced by PNP alone on biofilm architecture was approximately similar to control. aPDT treated cells showed reduction in the numbers of bacterial cells (Fig. 7B3,B4). Furthermore, the number of cells was found to give a more reduction when the biofilm was subjected to aPDT^plus^ (Fig. 7B5,B6).

### aPDT and aPDT^plus^ affect the biomass accumulation

In contrast to the response to PNP alone, biomass decreased under PhotoActive^+^- and TBO-aPDT compared to control (*P* = 0.368, and 0.061, respectively). Figure 7C shows that PhotoActive^+^- and TBO-aPDT^plus^ produced a larger reduction of biomass weight than in either PhotoActive^+^- and TBO-aPDT, respectively (*P* = 0.017, and 0.010, respectively).

### aPDT and aPDT^plus^ reduced *S. mutans* metabolic activity

Although considerable reduction of metabolic activity of *S. mutans* was observed upon exposure with PhotoActive^+^- or TBO-aPDT, however, therapy employing PhotoActive^+^- or TBO-aPDT^plus^ produced a marked reduction of metabolic activity of *S. mutans*. The metabolic activity of *S. mutans* after treatment with PNP at a concentration of 12.5 mg/mL and PhotoActive^+^- or TBO-aPDT was decreased to 30.00, 75.63 and 83.51%, respectively (*P* = 0.041, 0.005 and 0.003, respectively; Fig. 7D), whereas treatment with PhotoActive^+^- and TBO-aPDT^plus^ rendered a more significant reduction of metabolic activity of *S. mutans* (86.58 and 89.77%, respectively; both *P* = 0.003; Fig. 7D).

### Monitoring of *gtfB*, *gtfC* and *ftf* genes expression from *S. mutans* versus aPDT and aPDT^plus^

**Figure 8:**
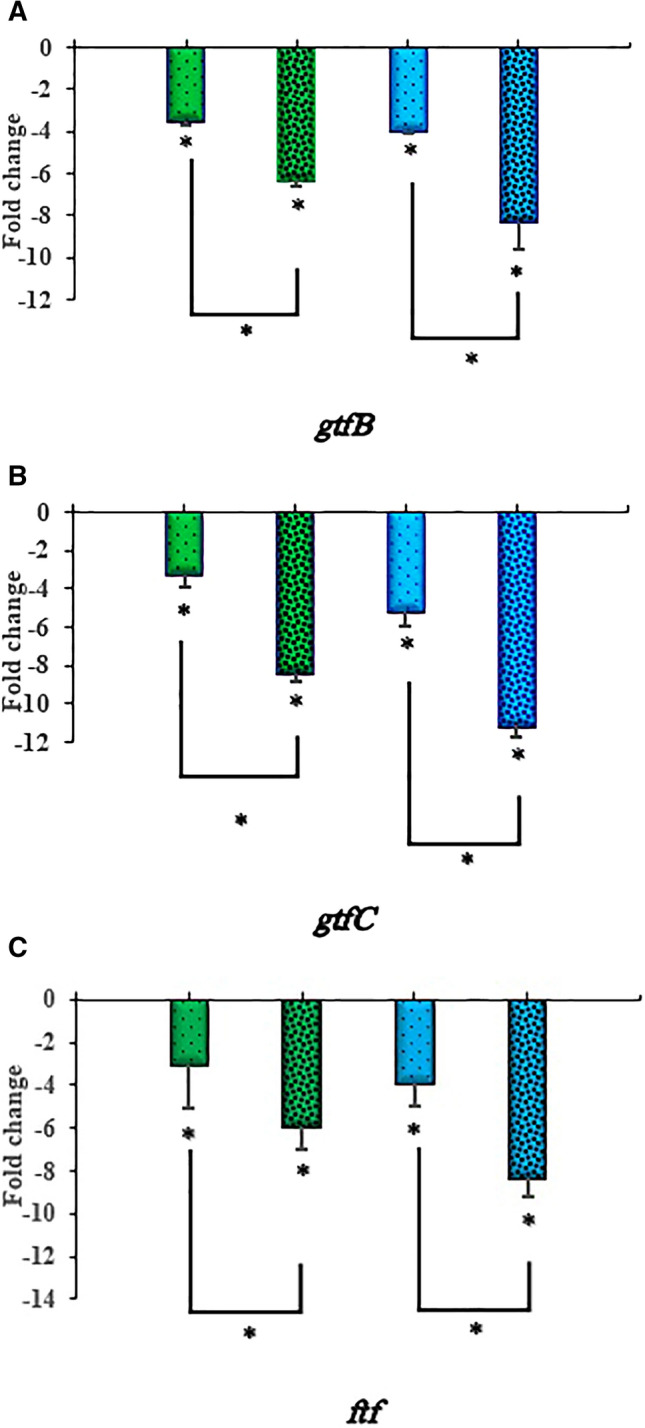
Quantitative real-time PCR analysis of genes involved in biofilm formation of *Streptococcus mutans*. (**A**) Expression profile of *gtfB* gene of *Streptococcus mutans* in response to the treatment with PhotoActive^+^- and TBO-aPDT and PhotoActive^+^- and TBO-aPDT^plus^; (**B**) expression profile of *gtfC* gene of *Streptococcus mutans* in response to the treatment with PhotoActive^+^- and TBO-aPDT and PhotoActive^+^- and TBO-aPDT^plus^; (**C**) expression profile of *ftf* gene of *Streptococcus mutans* in response to the treatment with PhotoActive^+^- and TBO-aPDT and PhotoActive^+^- and TBO-aPDT^plus^. Data are presented as means ± standard deviations. *p < 0.05.

To clarify the relationship between PNP and expression of biofilm-associated genes in *S. mutans* cells, qRT-PCR was employed. The *gtfB*, *gtfC* and *ftf* mRNA levels significantly decreased after exposure to PhotoActive^+^- and TBO-aPDT compared to control (*P* < 0.05). In particular, the expression level of the *gtfB*, *gtfC* and *ftf* genes in *S. mutans* cells treated with PhotoActive^+^- and TBO-aPDT^plus^ were higher than in treated *S. mutans* cells without PNP (*P* < 0.05). As shown in Fig. 8A–C, the expression of *gtfB*, *gtfC* and *ftf* were reduced by 3.53, 3.24, 3.01 and 4.00, 5.16, 3.86 fold following PhotoActive^+^- and TBO-aPDT, respectively. Moreover, the expression levels of *gtfB*, *gtfC* and *ftf* were reduced by 6.36, 8.45, 5.93 and 8.28, 11.15, 8.33 fold following PhotoActive^+^- and TBO-aPDT^plus^, respectively. Taken together, PNP is synergistic with PSs in decreasing the expression level of the biofilm-associated genes in *S. mutans* cells. A summary of the antimicrobial tests is given in Table 1.

**Table 1:** Summary table of the antimicrobial tests.

Antimicrobial tests II	Groups							
	Control	PNP	PhotoActive^+^	TBO	PhotoActive^+^-aPDT	TBO-aPDT	PhotoActive^+^ -aPDT^plus^	TBO-aPDT^plus^
MIC		25 mg/mL	625 μg/mL	12.5 μg/mL				
Checkerboard method (synergy)		6.2 mg/mL	156.2 μg/mL	1.5 μg/mL				
aPDT					PhotoActive^+^ **(**156.2 μg/mL) + Diode laser (103.12 J/cm^2^ )	TBO (6.25 μg/mL) + Diode laser (68.75 J/cm^2^ )		
aPDT^plus^		3.1 mg/mL					PhotoActive^+^ (156.2 μg/mL) + Diode laser (103.12 J/cm^2^ )	TBO (1.5 μg/mL) + Diode laser (68.75 J/cm^2^ )
Biofilm formation (%)	100	89.29			72.00	60.32	42.00	37.68
Metabolic activity (%)	100	70.00			24.37	16.49	13.42	10.23
Dry weight (mg)	10.5	10.0			8.5	4.5	3.0	1.0
*gtfB* expression ↓					3.53	4.00	6.36	8.28
*gtfC* expression ↓					3.24	5.16	8.45	11.15
*ftf* expression ↓					3.01	3.86	5.93	8.33

## Discussion

Selective removal management is increasingly suggested for the control of dental caries, with the aim of maintaining pulpal health and controlling the disease rather than relieving its symptoms^3^. aPDT is one of the upcoming therapeutic approaches against biofilm-mediated bacterial infections^11^. NPs-based combination therapy improved drug stability, solubility, the aPDT efficiency and penetration power compared to a pure drug solution^23,24^. Moreover, size- and shape-dependent traits of NPs provide a unique benefit for therapeutic aims^25^. It was also observed that PNP not only increased the antibacterial activity by providing a synergistic effect, but also demonstrated good compatibility with HRBCs and HGF cells, which is a pivotal factor for in vivo administration^26^. On the other hand, the proton electrochemical gradient across the membrane is required bacterial viability. Propolis constituents may enhance membrane permeability and cause the loss of membrane potential^27^. This disorganization might allow easier penetration by the PS, resulting in the detected in vitro synergy^28^. Fuchs et al.^29^, showed that the higher membrane instability improves the photosensitizing process.

This study used the checkerboard method to screen combinatorial doses of PSs and PNP for synergistic conditions. This procedure is based on the broth dilution method, which is standardized for clinical use^30^. The virulence of *S. mutans* is attributed to the elaboration of biofilms that protect the bacterium^31^. As reported previously, the threshold concentration of the PS required for significant photoinactivation of biofilms^32^. Cell wall composition, rate of growth, and the presence of polysaccharide inter-cellular adhesion in a biofilm differs greatly from planktonic lifestyle. Therefore, there is a difference in the photodynamic effects that may obstruct the uptake of the PS, as well as produces a dramatic decrease in light reaching the bacteria and thereby makes the cells less susceptible to aPDT^10,32^.In an in vitro study investigating the effectiveness of 100 μg/mL TBO mediated aPDT on *S. mutans* in biofilm form, a significant biofilm reduction of up to 63.87% was found^12^. In an earlier report, it has been shown that 5,000 μg/mL of PhotoActive^+^ is necessary to degrade 36.93% of *S. mutans* biofilms formed under exposure to sucrose on human enamel slabs^13^. The results of current study confirmed that the combination of PNP with PSs at lower concentration levels significantly boosted the antibacterial and antibiofilm activity against *S. mutans*. In addition, Fe-SEM images of biofilms formed after the selected treatments were assessed. PNP exposure alone induced a slight decrease in biofilm formation compared to aPDT^plus^. It is probably because propolis compounds could be destroyed in the bacterial suspension of hydrolytic enzyme activities^33^, while PhotoActive^+^- or TBO-aPDT treatment was smaller than in aggregates from control and a decrease in the number of cells could be detected. On the other hand, the reduced cell density and single bacterial cells or in pairs or short chains visualized following PhotoActive^+^- or TBO-aPDT^plus^ indicates the loss of cells within the biofilms. Therefore, these observations confirm that synergistic effect. These results are consistent with the results reported by Misba et al.^11^, which show the more substantial reduction of *S. mutans* biofilm mass following aPDT using TBO–silver NPs conjugates. The results obtained from the XTT assay against *S. mutans* confirmed that the powerful antimicrobial activity of the PhotoActive^+^- or TBO-aPDT^plus^ compared to the each agent alone. Further investigation through affecting the gene expression of biofilm regulatory genes, including *gtf* genes and *ftf* were identified. A potent inhibitor of *gtf* genes is reported to be capable of reducing the amount of Gtfs in *S. mutans* biofilms, thereby inhibiting EPSs production and biofilm formation^34^. Munro et al.^7^ reported that inactivation of *gtfB, -C* or *ftf* drastically reduced virulence properties of *S. mutans*. When PhotoActive^+^- or TBO was exposed to its appropriate wavelength of light, a significant decrease in biofilm associated genes in *S. mutans* were found. However, on treatment with the PhotoActive^+^- or TBO-aPDT^plus^, a considerable down-regulation compared to the previous state was detected in all the examined genes. Ong et al.^21^, whose study demonstrate that the propolis affects gene expression and makes it more sensitive to treatment with antibiotics. Also, the results of the present study showed that *gtfC* was more affected in the combination‐treatment group than the other genes tested. Deletion of *gtfC* markedly reduces attachment to smooth surfaces by *S. mutans*^35^.

As mentioned previously, no study investigated the effects of PNP on increasing the potency of photodynamic treatment of caries. However, the efficacy of aPDT against bacteria in biofilm depends mainly on the penetration of the PS and light to the layer in the deeper parts of the biofilm. Based on the findings of this study, PNP can increase the efficacy of aPDT by scattering the biofilm structure and thereby enhancing the PS and light penetration. There are a number of limitations in the current study was that only varying concentration of PSs in synergism method was investigated. Since it usually requires high concentrations of PSs and high-energy doses of light to completely affect biofilm forming bacteria, different energy doses of light by varying power density and exposure time of diode laser should be assessed on bacterial biofilm models in future studies. It's also true that although the major virulence traits of *S. mutans* treated with PNP and PSs in vitro was investigated in the present study, in vivo studies will be the next step to confirm the therapeutic usefulness of PNP on aPDT.

## Conclusions

Taken together, the findings obtained in this study reveal that the PSs with minimum concentration enrichment with PNP can improve antimicrobial photodynamic activities to a statistically significant degree against *S. mutans* in biofilm life forms. Moreover, the individual dose of the PSs can also be decreased, and which may cause minimize in adverse effects of them. Although more studies are needed to determine the mechanism involved in it, this synergistic effect of PNP and PhotoActive^+^- or TBO-aPDT may lead to a new mode of aPDT-based treatments in localized infections.

## Materials and methods

### Bacterial strain and culture conditions

*S. mutans* (ATCC 35668) was obtained from the Iranian Biological Resource Center, Tehran, Iran. The bacterium was grown aerobically (5% CO_2_) overnight in brain heart infusion (BHI) broth (Laboratorios Conda, Torrejón de Ardoz, Spain) at 37 °C.

### PSs and light source

Stock solutions of PhotoActive^+^ (W Medical systems GMBH, Germany) and TBO (Sigma-Aldrich, Steinheim, Germany) at concentrations of 5.0 and 0.4 mg/mL, respectively were prepared in distilled water, filter sterilized (0.22 μm pore size) and kept in the dark conditions prior to use. Diode laser (Klas-DX62, Konftec, Taiwan) at wavelengths of 635 nm with a maximum output power of 220 mW was used for activation of PSs.

### Minimum inhibitory concentrations (MICs) of PSs

The MICs of PhotoActive^+^ and TBO were performed as mentioned in previous studies^13,36^. Briefly, for each PS, 100 μL of BHI broth was added to the well of a round-bottom 96-well microplate and 100 μL of each PS solution (5 mg/mL PhotoActive^+^, 0.4 mg/mL TBO) was added to the first well in column 1 and diluted 1:2 to column 10. Then the columns were inoculated with 100 μL/well of bacterial suspension (1.0 × 10^6^ CFU/mL). Column 11 contained the bacterial suspension as a positive control and column 12 contained BHI broth without inoculum was considered as the negative control. The microplates were incubated for 24 h at 37 °C. The MIC was described as that concentration of PSs that significantly reduced bacterial growth.

### aPDT

The PhotoActive^+^- and TBO-aPDT were determined as described in previous studies^13,36^. Briefly, 100 μL of PSs at 1/2 and 1/4 MIC concentrations were diluted in flat-bottom 96-well microplates according to the mentioned above. The wells were then inoculated with 100 μL/well bacterial suspensions (1.0 × 10^6^ CFU/mL). The microplates were incubated for 5 min in the dark and then irradiated to diode laser to yield the desired energy densities. The control group did not receive any treatment. Then, 10 μL of each concentration was serially diluted (1:10 to 1:1,000) and 10 μL of each suspension was transferred to BHI agar (Merck, Darmstadt, Germany). The microplates were incubated at 37 °C in 5% CO_2_ for 48 h. Subsequently, the CFU/mL was computed using the Breed et al. method^37^.

### Preparation of alcoholic extract of propolis

The raw propolis was collected from of honey bees located in Isfahan, Iran. It was crushed into fine powder using an electric mill. A 10% (w/v) extract of propolis was prepared after adding 85% ethanol at 37 °C for 48 h on a shaker at 150 rpm. Then, the liquid portion was filtered through Whatman No. 1 filter paper. The filtered solution was maintained at 4 °C for 24 h and filtered several times.

### Preparation of PNP

In order to obtain PNP, the resulting extract was dispersed into the aqueous phase. This suspension was sonicated for 20 min and then evaporated in a water bath at 50 °C to concentrate. To obtain a dried powder, a freeze-drying machine (Lyotrap/Plus, UK) was used.

### Characterization of PNP

PNP was characterized as described by Kazemi and co-workers^38^. PNP analyzed by Field emission scanning electron microscopy (Fe- SEM; HITACHI S-4160, Japan). Fe-SEM can help us to have more accurate evaluation of the size distribution of NPs. Also, in order to understand the size distribution and poly dispersity index of synthetized PNP, the tests of dynamic light scattering (DLS) measurement were performed. In addition, zeta-potential of PNP was performed at 25 °C by using a Horiba Scientific Nanoparticci (SZ-100) instrument.

### MIC of PNP

The MIC of PNP was determined by the micro broth dilution method described in the guideline of Clinical and Laboratory Standards Institute (CLSI, Wayne, PA, USA). Briefly, 100 μL of *S. mutans* cells (1.0 × 10^6^ CFU/mL) was incubated with PNP (stock solution = 200 mg/mL) that diluted two-fold in 100 μL of BHI broth from column 1 of 96-well round-bottomed microplates to column 10 (concentration range of 50–4.8 mg/mL). After incubations at 37 °C for 24 h under aerobic conditions with 5% CO_2_, MIC is determined based on the lowest concentration that inhibits bacterial growth.

### Time–kill assay of PNP

To find out the activity of PNP against *S. mutans*, this study performed a time-kill assay to determine the ideal incubation time^19^. Briefly, aliquots of bacterial suspension at a final concentration of 1.0 × 10^7^ CFU/mL exposed to PNP at sub-MIC levels for 0, 5, 10, 15, 20, 25, 30 min in an aerobic incubation with %5 CO_2_. Next, samples were serially diluted (1:10 to 1:1,000) and then 10 μL of each suspension plated onto BHI agar for 48 h at 37 °C in 5% CO_2_. The CFU/mL was calculated based on the method mentioned in “MIC of PNP”.

### Checkerboard method

Detection of a synergistic combination of PSs and PNP was performed according to Hsieh et al.^39^. Briefly, a double dilution matrix of PSs and PNP was constructed in a 96-well microplate by diluting the PNP across the X axis while individual PS was titrated across the y axis. Titration was performed at a concentration range of 2 × MIC to 1/8 × MIC by two-fold serial dilution in BHI broth. Wells were inoculated with 100 μL of bacterial suspension (*S. mutans*, 1.0 × 10^6^ cells/mL). Synergy was evaluated by calculating the sum of the fractional inhibitory concentration index (FICI) as follows: FICI = FICA + FICB = ($${\raise0.7ex\hbox{${{\text{MIC A}} + {\text{B}}}$} \!\mathord{\left/ {\vphantom {{{\text{MIC A}} + {\text{B}}} {\text{MIC A}}}}\right.\kern-\nulldelimiterspace} \!\lower0.7ex\hbox{${\text{MIC A}}$}}$$) + ($${\raise0.7ex\hbox{${{\text{MIC A}} + {\text{B}}}$} \!\mathord{\left/ {\vphantom {{{\text{MIC A}} + {\text{B}}} {\text{MIC B}}}}\right.\kern-\nulldelimiterspace} \!\lower0.7ex\hbox{${\text{MIC B}}$}}$$). The lowest cutoff value for a combination to be described synergistic was FICI ˂ 0.5.

### Time-kill assay of PhotoActive^+^ or TBO alone and in combination with PNP

Time-kill assay was performed as described by Leonard et al*.*^40^. Briefly, The *S. mutans* suspension was adjusted to 1.0 × 10^6^ CFU/mL. PSs were examined individually (at ½ × MIC) as well as in combination. At different time points (0, 4, 8, 12, and 24 h) the number of cells is counted by performing serial dilutions on an aliquot removed from the treated culture. Colonies were counted in CFU/mL after incubation for 24 h at 37 °C in 5% CO_2_. Synergy was defined as ≥ 2 log_10_ CFU/mL decrease in bacterial count with the combination, in comparison with the single agent tested after 24 h.

### aPDT^plus^

PhotoActive^+^ and TBO at sub-significant reduction of CFU/mL (156 and 1.5 μg/mL) and PNP at sub-MIC concentration (3.1 and 1.5 mg/mL) were used, respectively. Then, 100 μL from each PS poured to the corresponding wells of flat-bottom 96-well microplate and 100 μL of PNP added to them. For the combined therapy, aliquots of 100 μL of bacterial suspensions (1.0 × 10^6^ CFU/mL) were incubated with the PSs and PNP simultaneously for 5 min in the dark and then irradiated with a diode laser as mentioned earlier. Positive control was a combination of inoculum and BHI broth. Also, BHI broth without inoculum was used as negative control.

### Cytotoxicity assay

Human gingival fibroblast (HGF; IBRC C10459) was obtained from the Iranian Biological Resource Center (Tehran, Iran). The cells were seeded into 96-well plates at a density of about 10,000 cells per well in Dulbecco’s modified Eagle’s medium (DMEM; Biowest, France). Medium was supplemented with 10% fetal bovine serum (Gibco, UK) and pen-streptomycin (Biowest, France) in the presence of 5% CO2 at 37 °C.

After 24 h, the medium was replaced with 100 μL of PhotoActive^+^ (156.2 and 312.5 μg/mL), TBO (1.5, 3.1 and 6.25 μg/mL), and PNP at different concentrations (1.5, 3.1, 6.2, and 12.5 mg/mL). In addition, 100 μL of each PS alone and in combination with PNP was added into the wells and irradiated with diode laser to yield the desired energy densities. The reaction solution was then readily removed and 100 μL of fresh medium was added to each well. Control cells without tested compound were incubated with a fresh culture medium. Following incubation for 24 h, a 3-(4,5- dimethylthiazol-2-yl)-2,5-diphenyltetrazolium bromide (MTT) reagent was added to each well and incubated for 4 h. Finally, the incubation solution was then removed and the formazan crystals were solubilized by addition of 150 μL/well of dimethylsulfoxide (DMSO, Merck). The absorbance of each well was measured at 570 nm by a microplate reader (Biotek Instruments, Inc.)^41^. Cell morphology was assessed by taking microscopy photographs in an inverted microscope (Olympus IX70, Tokyo, Japan). The permissible limit of cytotoxicity effect is considered to be > 75% according to ISO standards 10993-5:2009^42^.

### Hemolysis assay

The hemolysis assay was performed according to the method described by Li et al.^26^, with minor modifications. Briefly, fresh human blood was collected from a healthy adult female volunteer in Tehran, Iran. Human red blood cells (HRBCs) were centrifuged for 10 min at 1,000 rpm until the remove the supernatant plasma. Next, RBCs were purified 5 times through washing with PBS. The suspension was diluted in PBS buffer at a 1:10 ratio and then, 100 μL of the RBC suspension was separately added to 900 μL of PBS containing PNP at sub-MIC levels (1.5, 3.1, 6.2, and 12.5 mg/mL) and PhotoActive^+^ (156.2 and 312.5 μg/mL) or TBO (1.5, 3.1 and 6.25 μg/mL) alone, or in combination with PNP (3.1 mg/mL) and followed by mild shaking and kept for 2 h at room temperature (25 °C). Deionized water and PBS were used as positive and negative control, respectively. Later these samples were centrifuged (10,000 rpm, 1 min) and absorbance of the hemoglobin in the supernatants was measured by a UV–vis spectrophotometer (Alpha-1860, Shanghai Lab-Spectrum Instruments Co., Shanghai, China) at 541 nm. Images of the samples were taken using the mobile phone camera. The hemolytic percentages were calculated as follows the equation $$\left( {{\raise0.7ex\hbox{${{\text{A sample }}{-}{\text{ A negative control}}}$} \!\mathord{\left/ {\vphantom {{{\text{A sample }}{-}{\text{ A negative control}}} {{\text{A positive control }}{-}{\text{ A negative control}}}}}\right.\kern-\nulldelimiterspace} \!\lower0.7ex\hbox{${{\text{A positive control }}{-}{\text{ A negative control}}}$}}} \right) \, \times { 1}00$$.

### Biofilm formation assay

Following aPDT and aPDT^plus^, biofilm formation was evaluated by crystal violet method according to Borges et al.^43^ with some modifications. Briefly, after 48 h aerobic incubation with 5% CO_2_ at 37 °C, the wells were washed twice with PBS to remove unbanded cells. Afterward, the biofilms were fixed with methanol (Merck, Germany) for 15 min. Then microplate was emptied and dried at room temperature. Subsequently, 200 μL of 0.1% (w/v) crystal violet was added to each well for 15 min and then washed with PBS. Bound dye was released with 150 μL of 95% (v/v) ethanol. The absorbance at 550 nm was measured using a microplate reader (Thermo Fisher Scientific, US).

### Fe- SEM

This protocol was modified from Misba and co-workers^11^. Fe- SEM was performed in order to compare the effect of aPDT and aPDT^plus^ on a 48 h grown biofilm of *S. mutans*. Saliva was collected on ice from a healthy individual. It was centrifuged at 8,000 rpm for 15 min and then filter sterilized to obtain clear saliva. Then, 100 µl of cleaned saliva was added to each well from a 24-well microplate containing a Laser-Lok titanium disc with 10 mm diameter, 1 mm thickness and 8 μm grooves (BioHorizons, Birmingham, USA). The microplate was incubated at 37 °C for 2 h to coat the discs with salivary pellicle. After incubation, these discs were rinsed thrice with PBS. After treatment as described above, biofilm formation was initiated on the discs in the wells of a 24-well microplate. Afterward, the discs were washed with PBS to remove loosely attached cells. The biofilms were formed on discs were then fixed using 2.5% glutaraldehyde for 1 h at 4 °C. The samples were again washed thrice with PBS before treating them with 1% aqueous osmium tetroxide for 45 min. The discs were washed with PBS again and serially dehydrated. Finally, the samples were dried and before examination, they were placed on a mounting base and all were sputter-coated with a thin layer of gold. Samples were then investigated under a Fe- SEM.

### Biomass assay

Following aPDT and aPDT^plus^, biomass assay was evaluated by dry weight method^44^. At 48 h, the biofilm suspension was centrifuged (4,000 rpm, 20 min, 4 °C). The supernatant was discarded and the pellet was washed three times with PBS. The resulting pellet was dried in the dry oven at 105 °C, and weighed.

### XTT assay

The bacterial metabolic activity was determined by the reduction of sodium 3-[1-(phenylamino-carbonyl)-3, 4-tetrazolium]-bis (4-methoxy-6-nitro) benzene sulfonic acid hydrate (XTT Kit; Roche Applied Science, Indianapolis, IN, US) to a soluble formazan product^45^. Following aPDT and aPDT^plus^, 100 µL of the XTT solution was transferred to each prewashed well. The microplate was incubated for 4 h in the dark at 37 °C. Before each assay, fresh XTT solutions were prepared by mixing 5.0 mL XTT labeling reagent + 0.1 mL electron coupling reagent. The color formation was detected at 492 nm using a microplate reader. Wells without treatment considered as a control.

### Quantitative PCR analysis of *gtfB, gtfC* and* ftf* gene expressed by *S. mutans*

Evaluation of the *gtfB, gtfC* and *ftf* gene expression of *S. mutans* was completed after aPDT and aPDT^plus^ using quantitative real-time PCR (qRT-PCR). Total RNA extracted from the *S. mutans* using the RNX-plus solution (SinaClon, Iran) according to the manufacturer’s instructions. The purity and quality RNA was checked out with a NanoDrop spectrophotometer and agarose gel electrophoresis. After removal of genomic DNA by RNase-free DNase I treatment (Thermo Scientific GmbH, Deutschland, Germany), cDNA was synthesized by using the Revert Aid™ First Strand cDNA Synthesis kit (Fermentas) in a 20 μL reaction volume. RT-PCR was carried out with a SYBR Green qPCR Master Mix (Bimake, USA) using the Line-GeneK real-time PCR detection system and software (Bioer Technology, Hangzhou, China). The RT‐PCR cycling conditions were as follows: 95 °C for 3 min.; then 40 cycles of denaturation at 95 °C for 15 s., annealing at 54 °C for 20 s., and extension at 72 °C for 30 s. The expression levels of target genes were analyzed through Eq. 2^-ΔΔCt48^. A sequence of primers applied in this work is shown in Table 2.

**Table 2:** The nucleotide sequences of the primers applied in this study.

Target gene	Oligonucleotide sequence 5′–3′	Amplicon size (bp)	References
*gtfB*	For. TGTTGTTACTGCTAATGAAGAA Rev. GCTACTGATTGTCGTTACTG	103	This study
*gtfC*	For. GAGTTGGTATCGTCCTAAGT Rev. CTGGTTGCTGTATTGTATGTT	177	This study
*ftf*	For. ACGGCGACTTACTCTTAT Rev. TTACCTGCGACTTCATTAC	98	This study
*16S rRNA*	For. GCAGAAGGGGAGAGTGGAAT Rev. GGCCTAACACCTAGCACTCA	182	Ref.^36^

### Statistical analysis

The results were obtained from three independent experiments. Differences between the groups were statistically analyzed by one-way analysis of variance (ANOVA) with the Tukey HSD post hoc test, and *p* < 0.05 was considered to indicate statistical significance. The commercial software SPSS version 23.0 was applied for all analyses.

### Ethical conduct of experiments

Permission from the Ethics Committee of Tehran University of Medical Sciences was received before commencing the experiments (IR.TUMS.MEDICINE.REC.1398.125). All experiments presented were performed in accordance with relevant protocols approved by the Tehran University of Medical Sciences (Protocol approval. no.: 98-01-30-41822). All individuals who agreed to take part in the study signed informed consent forms prior to enrolment in this research.
